# Supplementary figures and images for: Differential cell survival outcomes in response to diverse amino acid stress
Source: Life Sci Alliance. 2025 Sep 5;8(11):e202503324. doi: 10.26508/lsa.202503324 (PMC12413550; doi:10.26508/lsa.202503324)

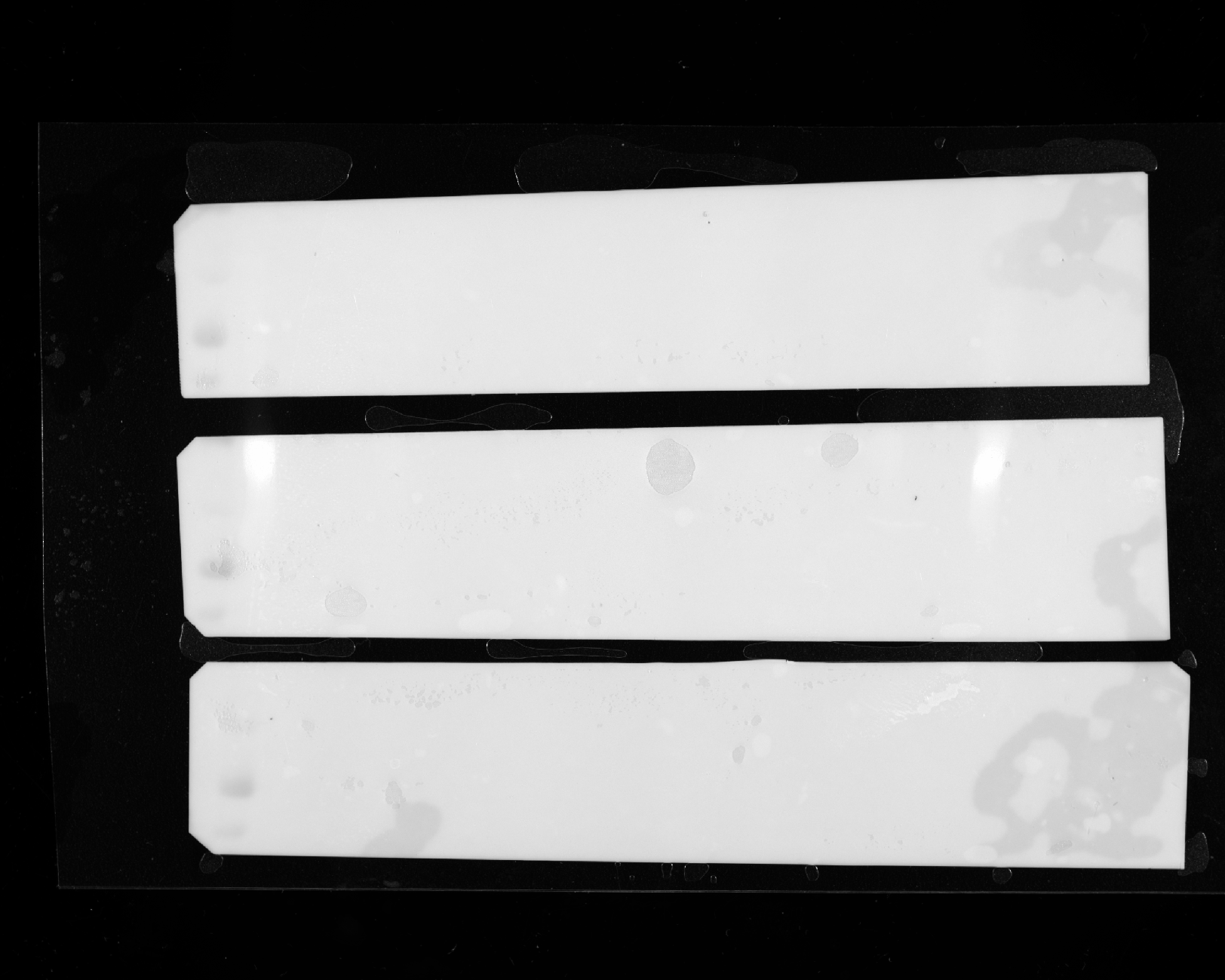

Supplement: Supplementary file 1 [file LSA-2025-03324_SdataF1.1.tif]

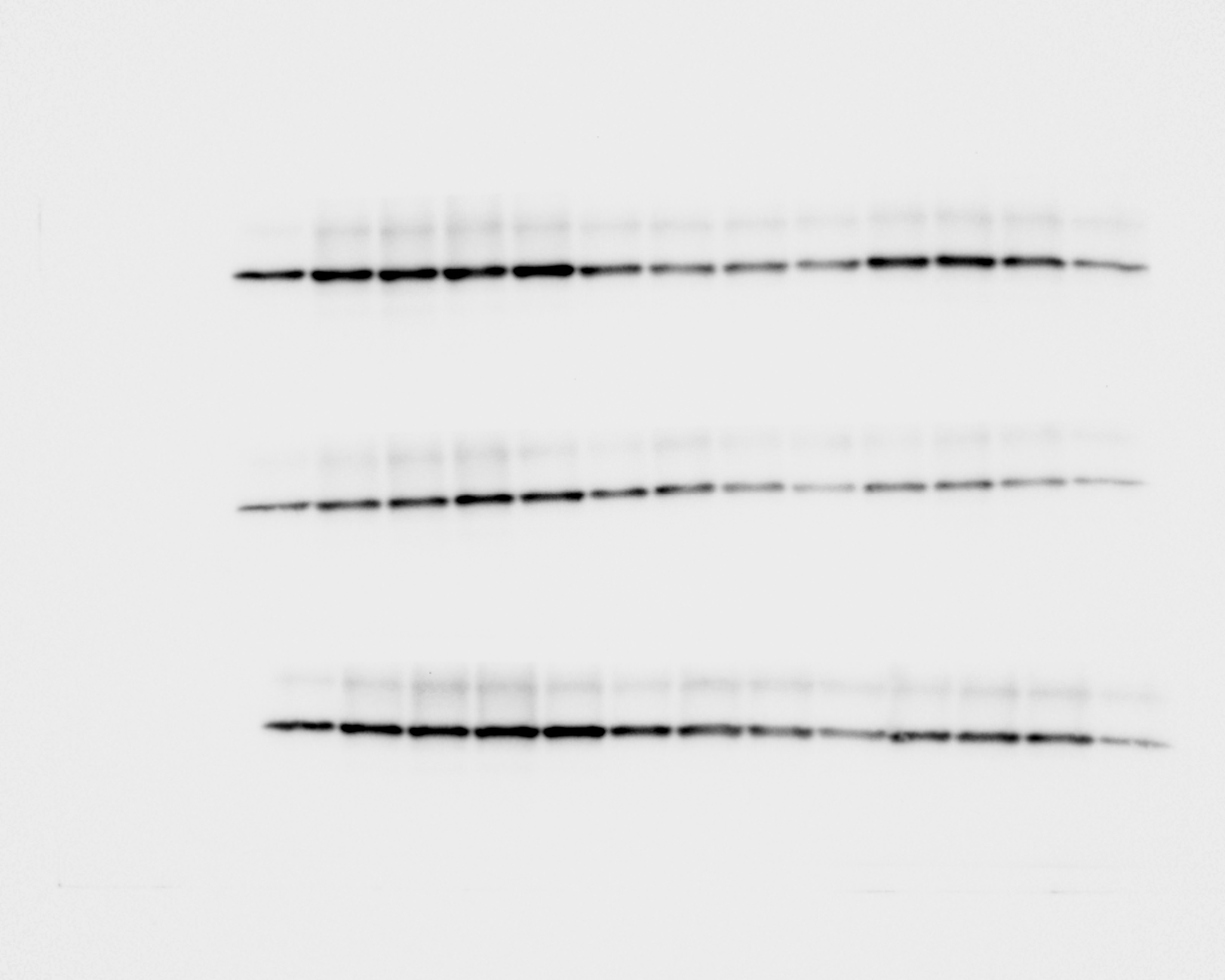

Supplement: Supplementary file 2 [file LSA-2025-03324_SdataF1.2.tif]

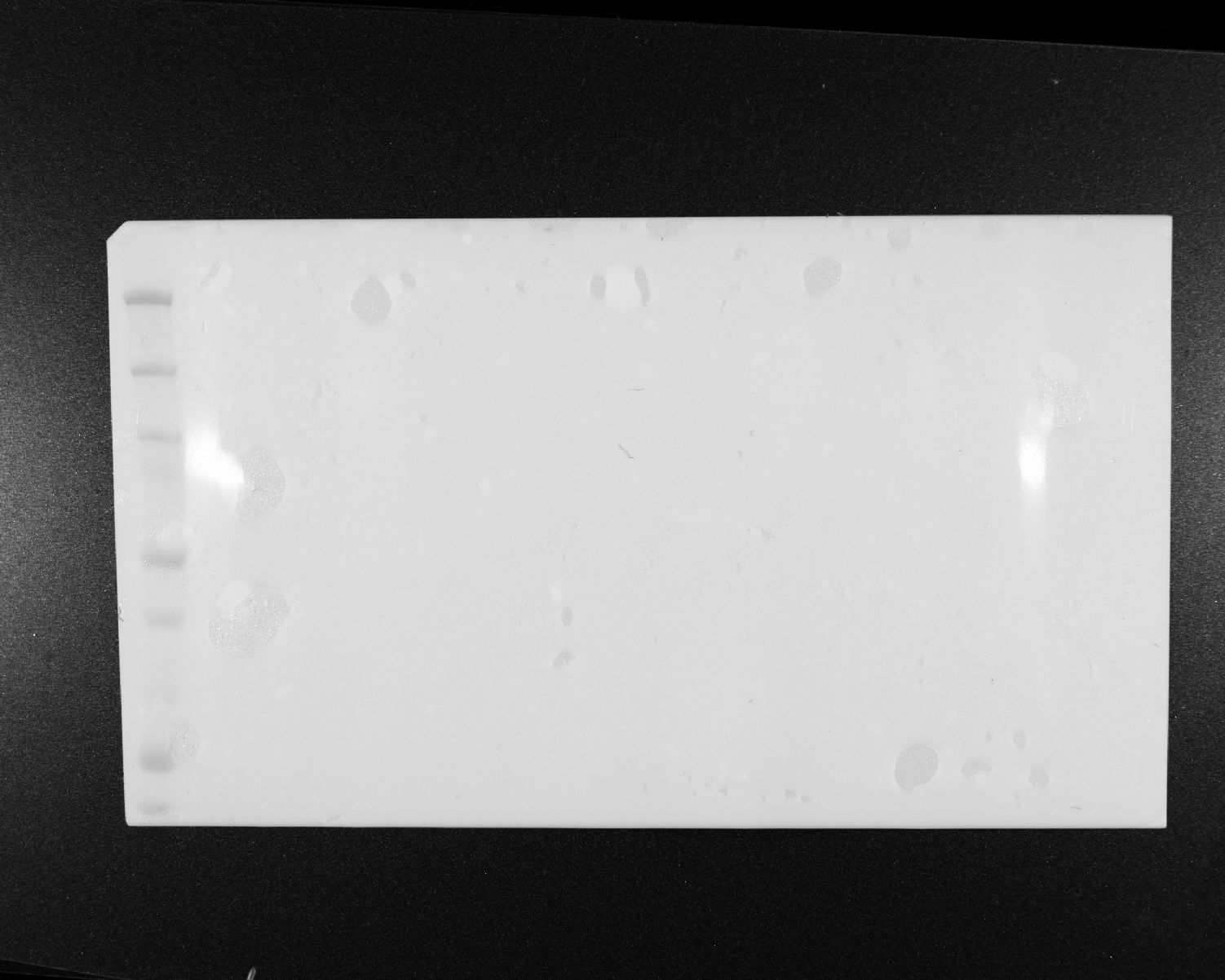

Supplement: Supplementary file 3 [file LSA-2025-03324_SdataF1.3.tif]

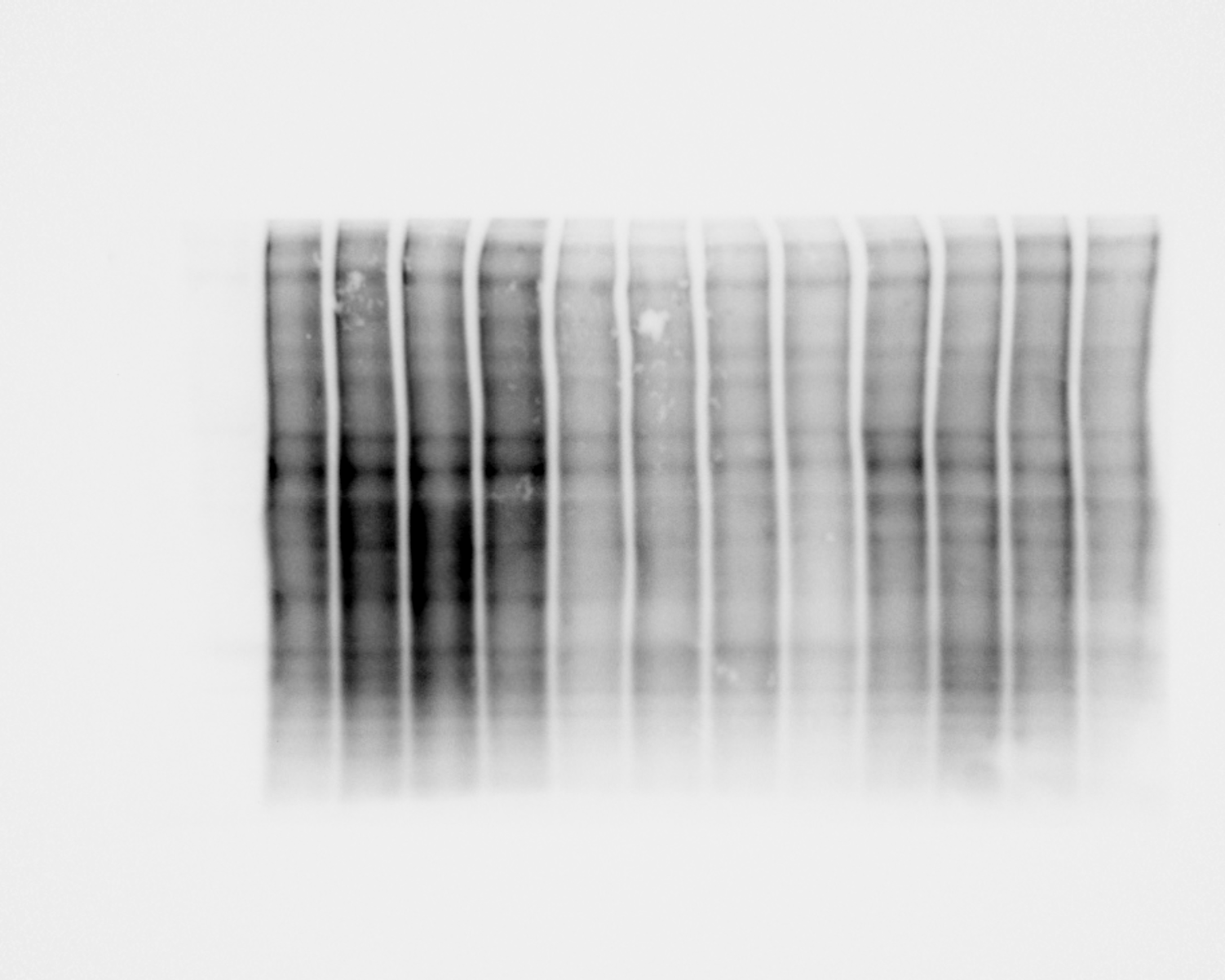

Supplement: Supplementary file 4 [file LSA-2025-03324_SdataF1.4.tif]

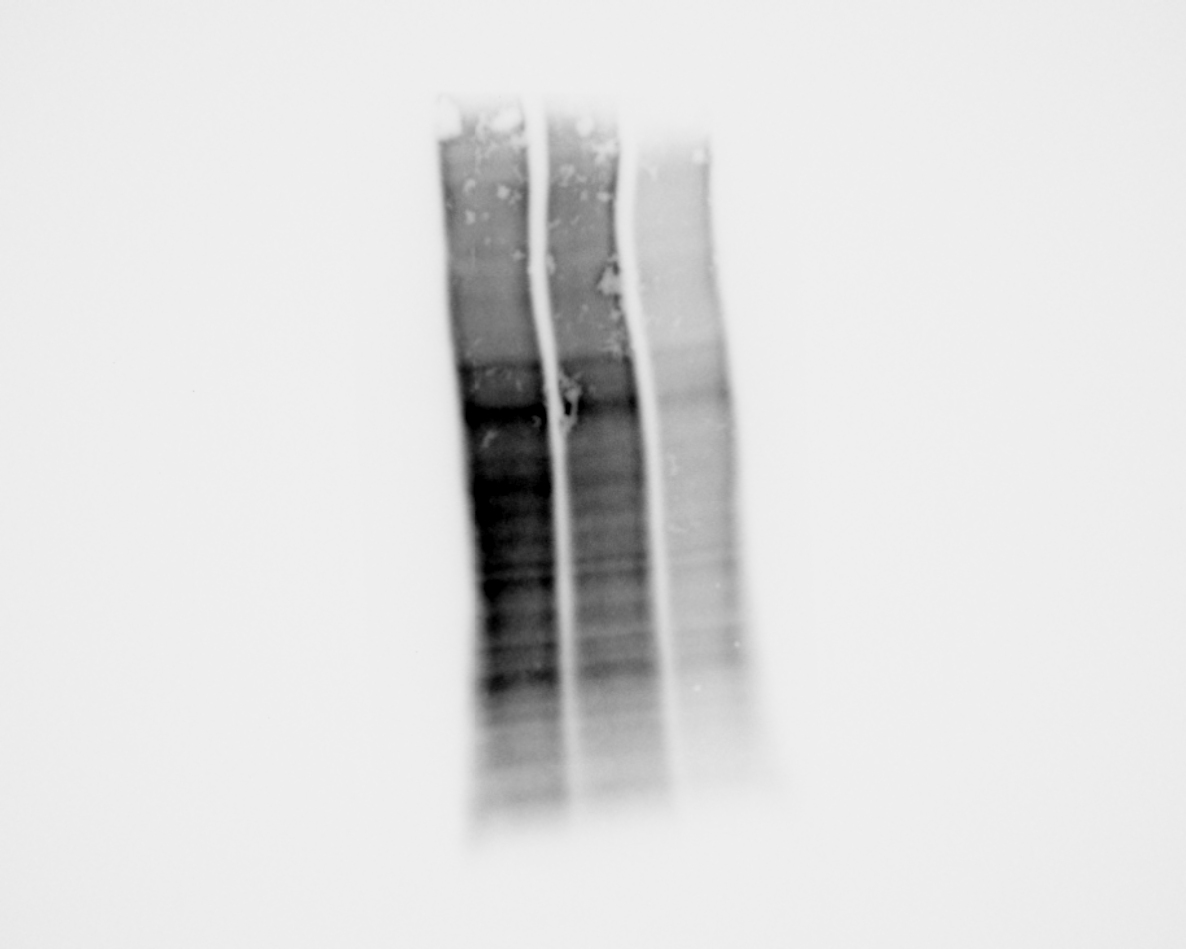

Supplement: Supplementary file 10 [file LSA-2025-03324_SdataF4.1.tif]

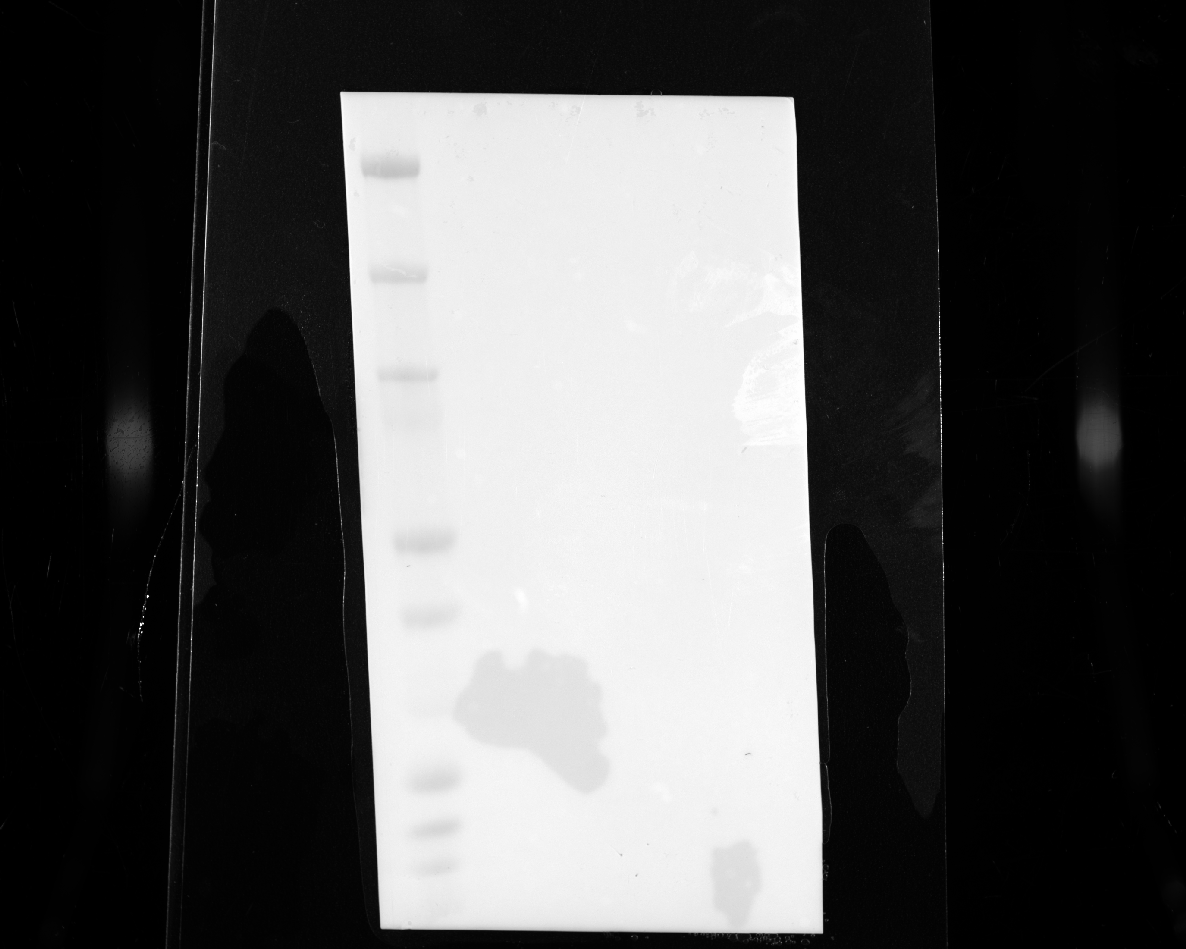

Supplement: Supplementary file 11 [file LSA-2025-03324_SdataF4.2.tif]

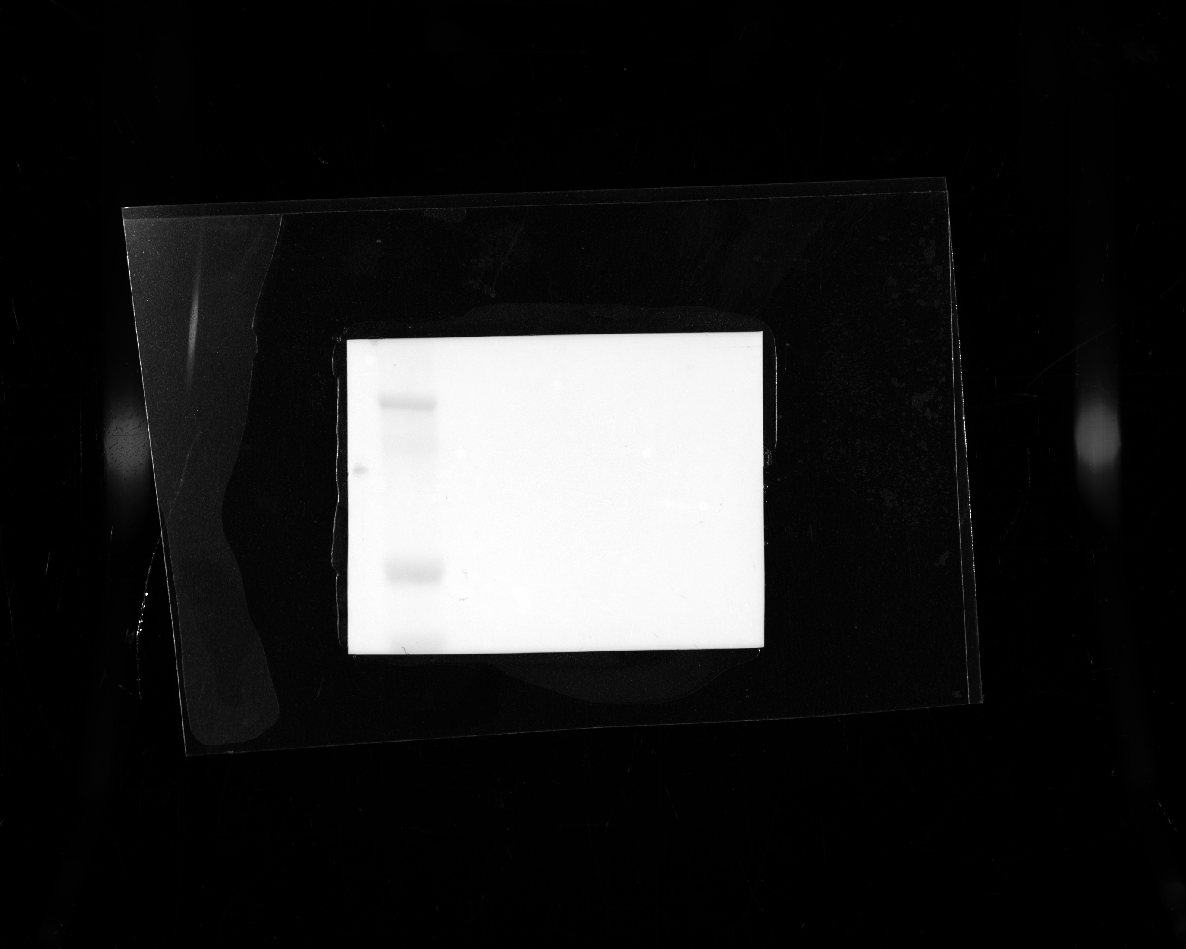

Supplement: Supplementary file 12 [file LSA-2025-03324_SdataF4.3.tif]

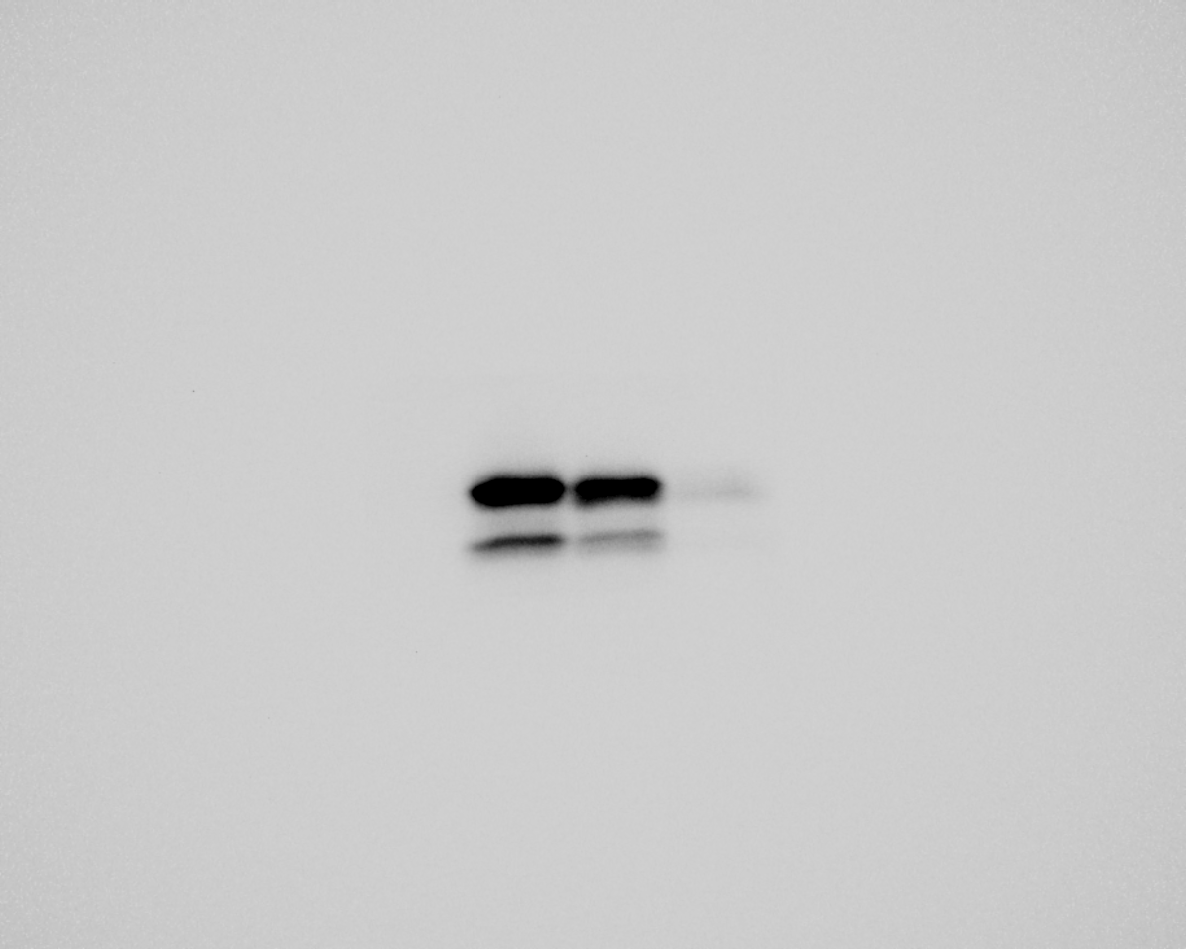

Supplement: Supplementary file 13 [file LSA-2025-03324_SdataF4.4.tif]

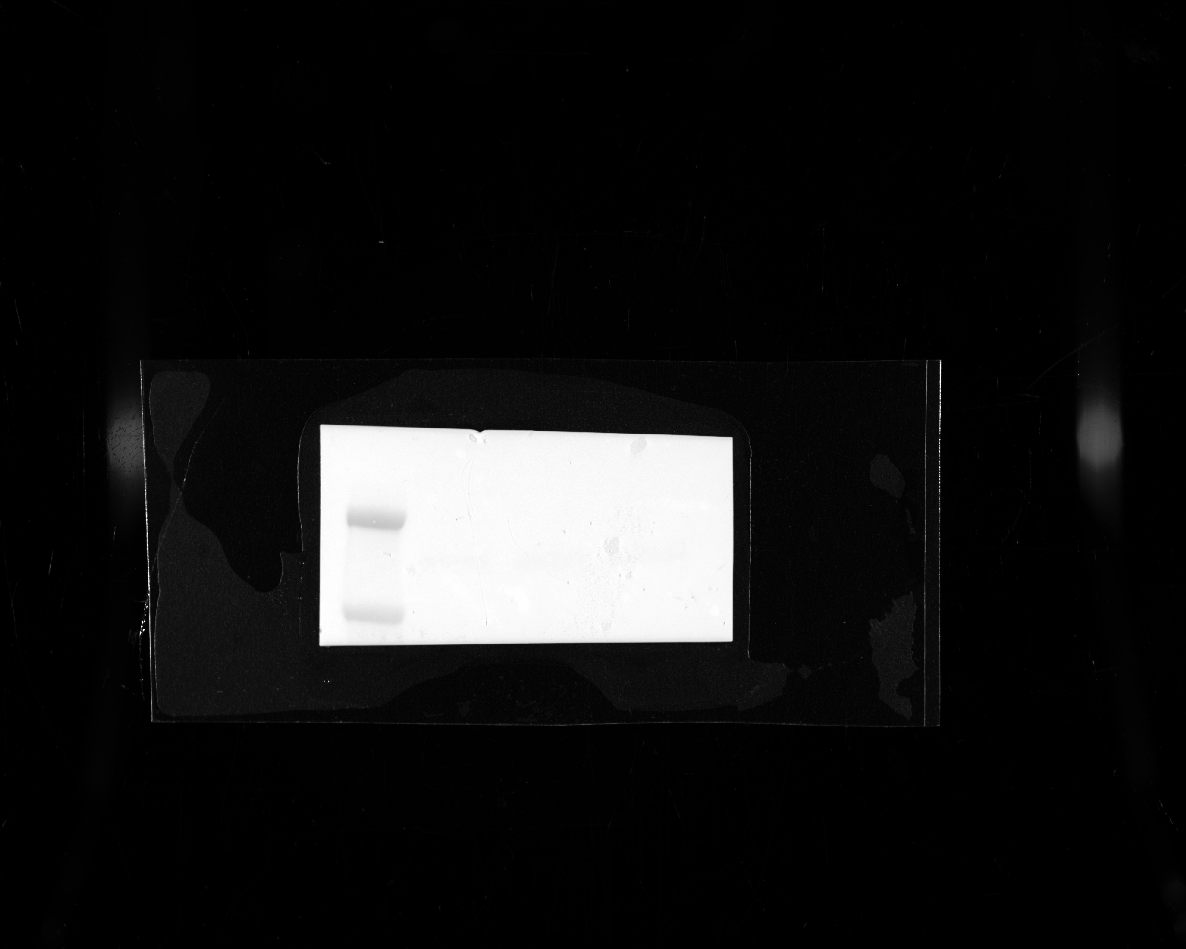

Supplement: Supplementary file 14 [file LSA-2025-03324_SdataF4.5.tif]

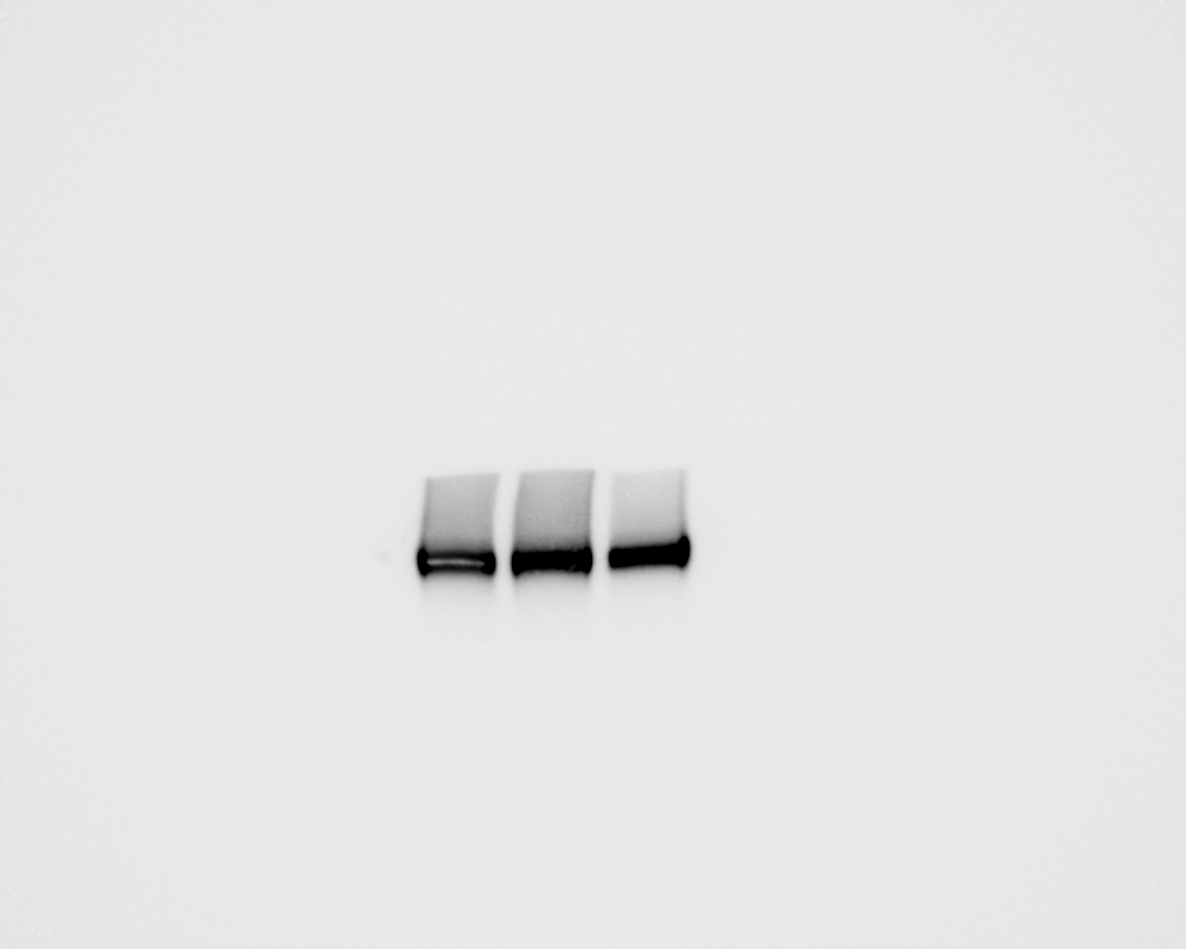

Supplement: Supplementary file 15 [file LSA-2025-03324_SdataF4.6.tif]
